# Quantification of lead through rod-shaped silver-doped zinc oxide nanoparticles using an electrochemical approach

**DOI:** 10.3762/bjnano.16.33

**Published:** 2025-03-26

**Authors:** Ravinder Lamba, Gaurav Bhanjana, Neeraj Dilbaghi, Vivek Gupta, Sandeep Kumar

**Affiliations:** 1 Department of Physics, Guru Jambheshwar University of Science and Technology, Hisar-Haryana, 125001, India; 2 Department of Bio and Nano Technology, Guru Jambheshwar University of Science and Technology, Hisar-Haryana, 125001, India; 3 Department of Physics, Punjab Engineering College (Deemed to be University), Chandigarh, 160012, India

**Keywords:** electrochemical methods, chemical sensor, doping, lead, nanoparticles, ZnO nanorods

## Abstract

Special features of zinc oxide nanoparticles have drawn a lot of interest due to their wide bandgap, high surface area, photocatalytic activity, antimicrobial activity, and semiconductor properties. By doping ZnO nanoparticles with transition metals, we can alter their electrical, optical, and magnetic properties by introducing new electronic states into the band structure. Herein, Ag is added to ZnO nanostructures to improve their optical properties to detect heavy metal lead ions. The prepared lead sensor with ultrahigh sensitivity, based on silver-doped ZnO nanorods (Ag@ZnO NRs), was fabricated and characterized. The morphological, structural, compositional, and optical characteristics of the Ag@ZnO NRs were investigated using a variety of methods after they were fabricated using a low-temperature co-precipitation method. The resulting Ag@ZnO NRs had good optical properties, nanorod morphologies, and high crystallinity with no impurities. Technological advancements are leading people to use lightweight electronics and affordable sensors. Electrochemical techniques comparatively offer quick, portable, sensitive, and inexpensive basic equipment for heavy metal detection. The interactions between Ag@ZnO NRs and lead were studied using electrochemical methods. The prepared lead sensor using Ag@ZnO NRs show a very low detection limit and a very high sensitivity toward lead. The lead chemical sensor that was developed had a detection limit of 3 ppm with a sensitivity of 16 µA·ppm^−1^·cm^−2^. The recorded reaction time of lead sensor was less than two seconds.

## Introduction

According to the literature, a lot of work has been done to create durable and dependable smart sensors for the effective identification of analytes that are harmful but also crucial for the environment and technology [1]. Even at very low levels, heavy metals can permanently harm health, and their acute exposure leads to chronic disorders by affecting organ functions [2]. For example, lead poisoning in liquid effluents, is one of the worst environmental hazards which can affect human health and readily impact immune responses [3]. Due to the detrimental effects on the environment and human health, determining the presence of trace heavy metals is crucial. Lead is a highly toxic element that affects human soft tissues and organs, acting in concert with other carcinogens to cause cancer in the kidneys, lungs, or brain. Lead paint, lead-containing petrol, mining, and smelting are some of the sources of lead exposure [4]. Through contaminated food or drink or through mouth-to-mouth contact, lead can enter the body. Standard techniques for determining lead content involve the use of atomic absorption spectroscopy (AAS) [5] and inductively coupled plasma–mass spectrometry (ICP–MS) [6]. Although both techniques yield reliable results in matrices as complicated as serum or blood, they require costly, large-scale equipment as well as highly skilled operators. Furthermore, these methods are not as desired or even appropriate for point-of-care (POC) use because of the considerable time delays caused by the shipment of samples to centralized laboratories. Miniaturization is made possible by electrochemical procedures for determining heavy metals, which need a comparatively simple apparatus. Thus, it is imperative to develop low-cost, miniaturized analytical instruments to monitor hazardous chemical substances [7–8]. Target detection in real time is a strong suit for electrochemical devices. Electron mediators are typically used to modify the working electrodes in electrochemical sensor fabrication. These days, due to their unique electrical and optical characteristics, nanomaterials are employed as effective electron mediators [9].

Zinc oxide nanoparticles have gained a lot of attention due to their unique features, such as wide bandgap (approximately 3.37 eV), excellent electron transportation, piezoelectric behavior, semiconductor nature, low toxicity, and enhanced electrochemical response, and have a vast range of uses. Zinc oxide shows excellent features, such as nanoscale particles, highly crystalline nature, tunable shape, size and density, and a high aspect ratio. In summary, ZnO nanoparticles offer a versatile platform for technological advancements across fields such as medicine, electronics, environmental remediation, and energy [10–11]. The use of certain metal dopants to modify the chemical, optical, and electrical features of a material has gained considerable interest in the realm of semiconductor technology. A recent study has conducted thorough investigations into the effects of transition metal ions, such as silver, copper, nickel, and manganese on the chemical and physical properties of ZnO nanoparticles. These metal dopants utilize their partially occupied d-electron shells, leading to the presence of unpaired electrons. Out of these metals, silver is particularly well-suited for ZnO doping because of its notable characteristics, including strong conductivity, solubility, favorable ionic size, and low orbital energy. These features contribute to the improvement of optical and electrical characteristics of ZnO. The incorporation of silver boosts the mobility of oxygen on the surface by means of the formation of oxygen vacancies, leading to enhanced catalytic activity. Also, the small doping of Ag introduces more active sites on the catalyst surface, potentially improving the overall catalytic activity [12–13]. This study demonstrates an efficient and uncomplicated method for producing Ag@ZnO NRs. These fabricated Ag@ZnO NRs play an effective role as an electron mediator in the development of lead chemical sensors which are both highly sensitive and robust in nature. Based on our current understanding, the lead sensor we developed exhibits the most notable sensitivity among all.

## Results and Discussion

### X-ray diffraction of as-synthesized Ag@ZnO nanorods

**Figure 1:**
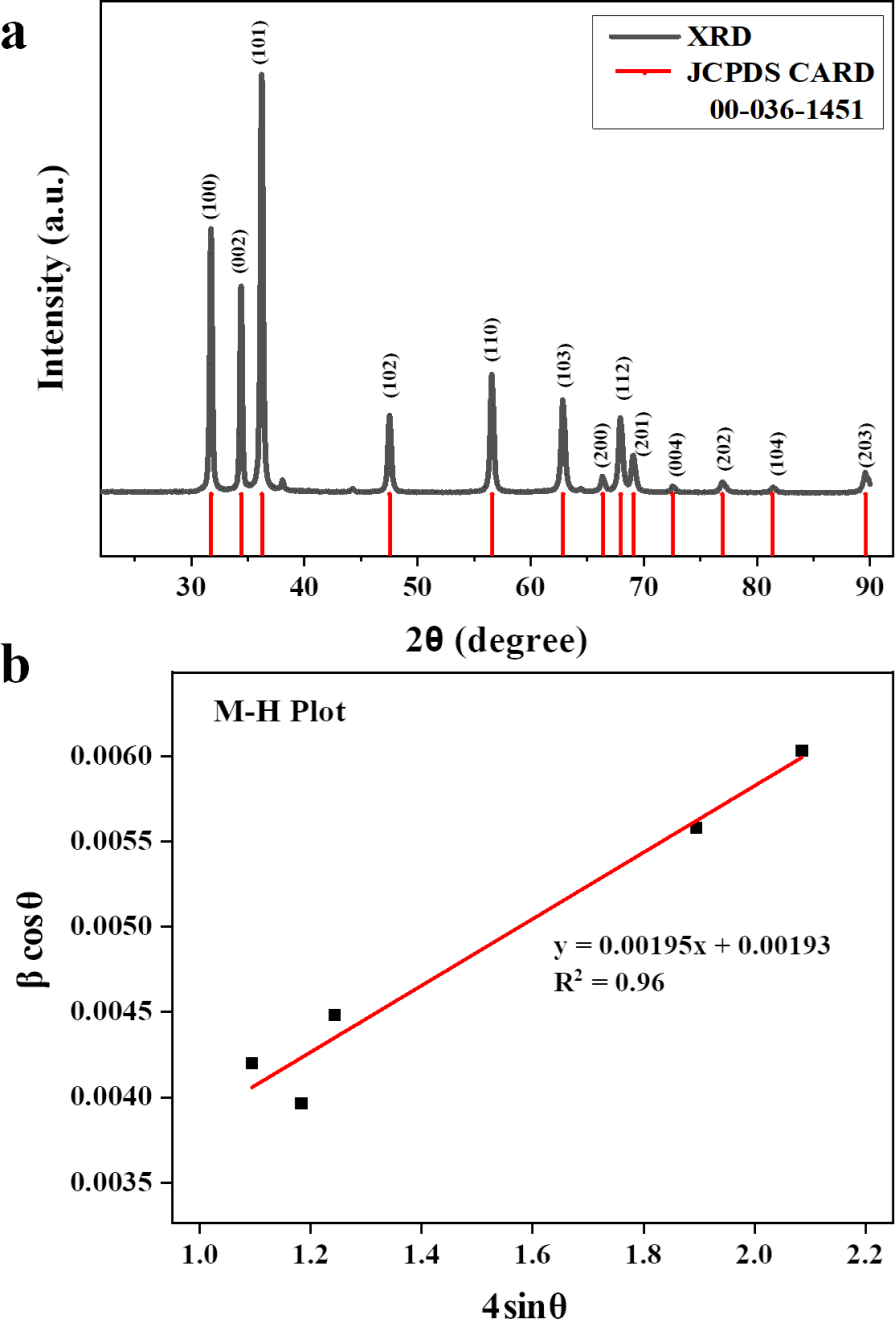
(a) XRD of Ag@ZnO NRs and (b) M–H plot of Ag@ZnO NRs.

The Ag@ZnO NRs were analyzed for their crystal phases by evaluating the X-ray diffraction pattern. Figure 1a displays the diffraction pattern of the Ag@ZnO NRs that were formed. It was observed that this pattern closely corresponds to the data that has already been reported [14]. The distinct reflections indicate the high degree of crystalline nature of the nanorods that were created. Ag@ZnO NRs have a hexagonal wurtzite structure with space group 186: *P*6_3_*mc*. The lattice is primitive with dimensions *a* = *b* = 0.32488 nm and *c* = 0.52001 nm. The peaks in this pattern are detected at specific angles, denoted as 2θ, which are 31.79°, 34.48°, 36.27°, 47.55°, 56.62°, 62.92°, 66.43°, 67.95°, 69.07°, 72.58°, 77.02°, 81.54°, and 89.69°. These angles correspond to the lattice planes (100), (002), (101), (102), (110), (103), (200), (112), (201), (004), (202), (104), and (203) respectively. The pattern of Ag-doped ZnO nanoparticles exhibits three additional diffraction peaks at 2θ values of 38.29°, 44.39°, and 64.58°. These peaks are associated with the metallic FCC phase of Ag. Ag doping at the substitution sites of the ZnO crystal lattice results in a decrement in the peak position values because Ag^+^ ion (12.2 nm) has a larger ionic size compared to that of the Zn^2+^ ion (7.4 nm). Consequently, the given data aligns with the decrease in the highest location of the point, indicating a reduction in the *c*-axis lattice. This implies that the Ag ion has filled the spaces between the atoms in the ZnO structure. Also, Ag functions as an amphiprotic dopant, meaning it can both donate and accept protons. It tends to occupy interstitial sites and also substitute for Zn [15–16]. Debye–Scherrer’s relation is used to calculate the average crystallite size of the Ag@ZnO NRs:




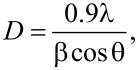




where λ = 1.54 Å (wavelength of the Kα radiation of Cu), β = full width at half maximum, and θ = peak position. The average crystal size of Ag@ZnO NRs is found to be approximately 28 nm.

The dislocation density (δ) of the crystalline material expressed as 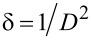 is 0.001275. The total broadening of the peak (β_T_) caused by the crystalline size and strain in the lattice is given by:




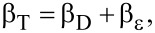




where β_D_ is broadening of the peak due to crystal size and β_ε_ is broadening due to lattice strain. For analyzing stress and strain resulting from X-ray diffraction, Williamson and Hall's approach is the simplest method. The peak broadening resulting from the lattice strain can be obtained by the Stokes–Wilson relation:




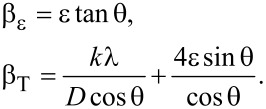




By plotting β_T_cosθ as a function of 4sinθ as shown in Figure 1b, we get the value of ε = 0.00195, and the intercept = kλ/*D* = 0.00193.

This plotted straight line using a W–H plot is a good fit as the correlation coefficient value of *R*^2^ is 0.96. Using the value of the intercept, the calculated size of the nanoparticle is found to be 71.8 nm. The value of the crystalline size obtained by the Williamson–Hall method is 2.5 times than that obtained by the Scherrer method. This difference is proportional to the strain value [17].

### Field-emission scanning electron microscopy of Ag@ZnO nanorods

**Figure 2:**
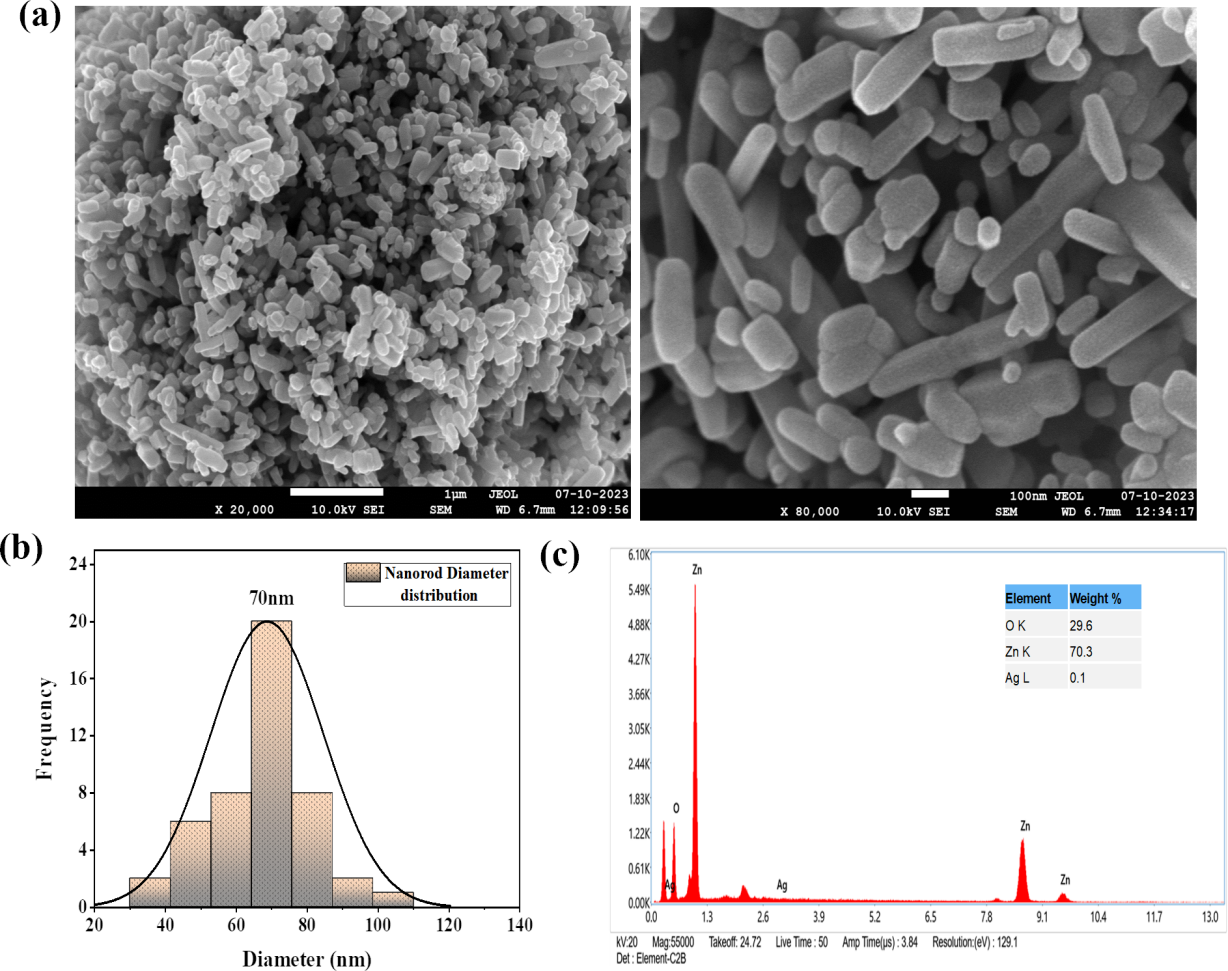
(a) SEM images, (b) diameter distribution of nanorods, and (c) EDS of Ag@ZnO NRs.

The general morphological characteristics of the as-obtained nanorods were analyzed by electron microscopy. Figure 2a depicts the typical field-emission scanning electron microscopy (FESEM) image of the as-obtained nanomaterials. The produced nanomaterials had rod-shaped morphologies and were grown at extremely high densities, as seen by the SEM image. Figure 2b represents the average diameter of Ag@ZnO NRs which was calculated using the Image J software. The average diameter of Ag@ZnO NRs is approximately 70 nm. The elemental composition of the fabricated nanorods was examined through energy-dispersive spectroscopy (EDS). Figure 2c depicts the typical EDS spectrum of the produced Ag@ZnO NRs. Observations from the EDS spectrum lead to the conclusion that the nanorods are made of zinc and oxygen. The produced nanorods are pure Ag@ZnO NRs with no detectable impurities, as evidenced by the absence of any other peak in the spectrum associated with any impurity.

### Fourier-transform infrared spectroscopy analysis of Ag@ZnO nanorods

**Figure 3:**
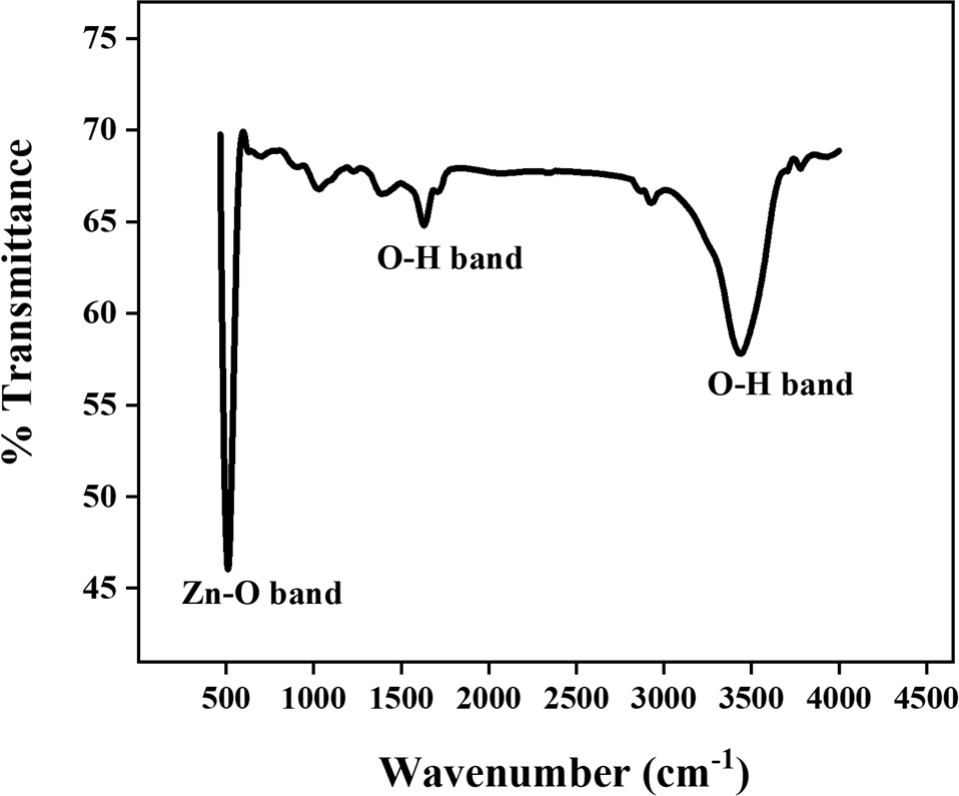
FTIR of Ag@ZnO NRs.

Figure 3 presents the typical Fourier-transform infrared spectroscopy (FTIR) spectrum of produced Ag@ZnO NRs. Different groups and bonds were examined using the FTIR spectrum. Ag doping changes the bond length of the sample, resulting in a minor shift in peak location toward higher wavelengths.

The distinctive stretching mode of the Zn–O bond is responsible for a significant vibrational band in the FTIR spectra, ranging from 450 to 550 cm^−1^. The presence of a hydroxy residue, which is caused by ambient moisture, is indicated by a large peak at 3433 cm^−1^ (stretching) and at 1330 to 1720 cm^−1^ (bending). Peaks present at 2922 and 2854 cm^−1^ relate to the stretching vibration of carbon and hydrogen bonds. The peaks at 1030 and 1380 cm^−1^ are related to vibrational or in-plane bending of residual ethanol which was used for washing the nanoparticles and KBr pellet die set (used for pellet making for FTIR) [18–19].

### Optical study of Ag@ZnO nanorods

**Figure 4:**
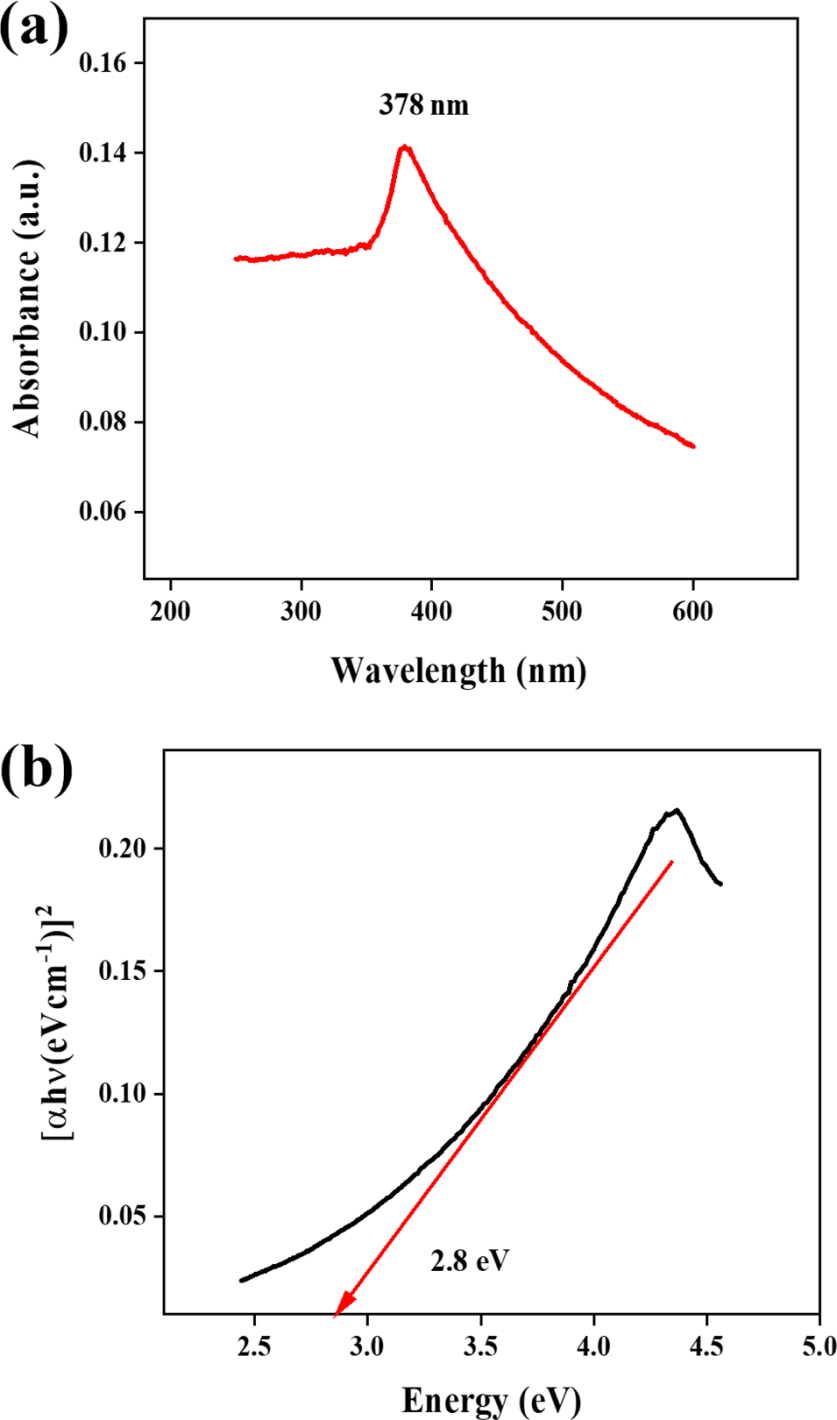
(a) UV–vis spectrum and (b) bandgap of Ag@ZnO NRs.

Figure 4a displays the optical spectra of Ag@ZnO NRs, which was obtained in the 200–600 nm wavelength range. The absorbance peak in this spectrum, which is moved toward a higher wavelength also known as redshift, is shown at 378 nm (for pure ZnO it is 362 nm) [20]. The bandgap energy of Ag@ZnO NRs has been calculated through the use of the Tauc's method, as shown in Figure 4b, and it is determined to be 2.8 eV.

No further peak indicative of contaminants is observed in the aforementioned spectra, indicating the high purity of the synthesized nanorods. Incorporating Ag dopants into ZnO frequently causes a reduction in the bandgap, leading to a shift toward longer wavelengths in the absorption spectra. The smaller bandgaps of the samples in optoelectronic devices provide a significant advantage [21].

### Zeta potential of Ag@ZnO nanorods

**Figure 5:**
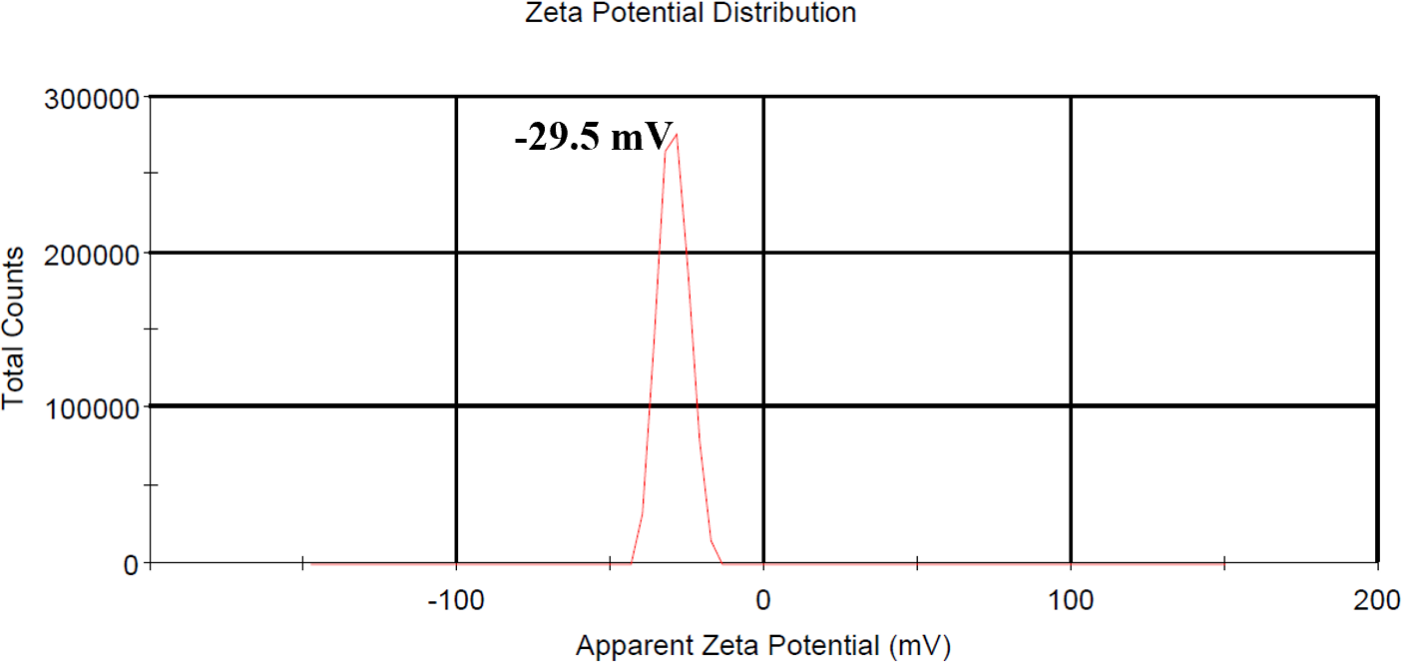
Zeta potential of Ag@ZnO NRs.

Surface properties of the synthesized Ag@ZnO NRs were studied using dynamic light scattering analysis, and their zeta potential was determined. Figure 5 represents the zeta potential of Ag@ZnO NRs. The samples were collected in the liquid state and the Ag@ZnO NRs zeta potential of ≈30 mV accounts for the stability of the nanoparticles in water.

### Raman spectroscopy of Ag@ZnO nanorods

**Figure 6:**
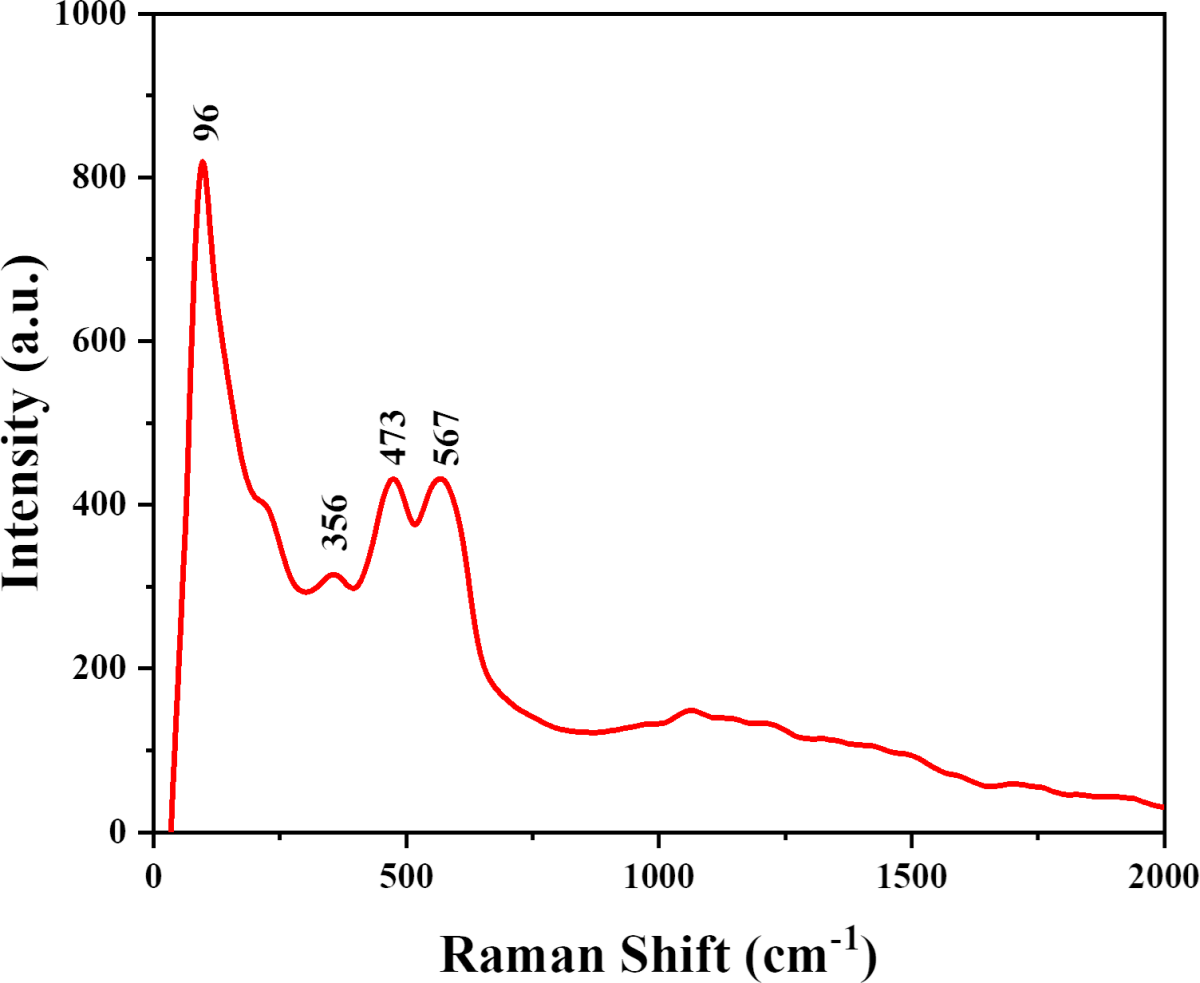
Raman spectra of Ag@ZnO NRs.

The influence of Ag doping in ZnO nanorods were investigated by Raman scattering. Raman scattering of Ag@ZnO NRs were recorded using a 532 nm laser at room temperature in the spectra range varying from 0 to 2000 cm^−1^. ZnO has four atoms in each primitive cell, which results in 12 degrees of freedom (three acoustic phonon modes and nine optical phonon modes). Figure 6 demonstrates the Raman spectra of Ag@ZnO NRs; when doping is done in ZnO, there is a significant change in the optical and non-optical modes of ZnO. The collapse of the translational crystal symmetry is a consequence of Ag doping, which also results in peaks broadening. The wurtzite structure of ZnO is characterized by dominant peaks at approximately 96 and 473 cm^−1^, the peak at 473 cm^−1^ represents the E_2H_ mode corresponding to oxygen, and this mode is sensitive to internal stress. Ag doping results in the broadening of the E_2H_ mode and also expands and relocates the E_1_(LO)/A_1_(LO) peak, present at approximately 567 cm^−1^, to a lower energy level. The broadening and shifting of the A_1_(LO) modes is the result of the scattering effects generated by the A_1_(LO) branch extending beyond the central region of the Brillouin zone. Oxygen vacancies are commonly linked to the A_1_(LO) phonon mode. The presence of a small peak at 218 cm^−1^ denotes the radial movement of Ag atoms. Raman peaks at around 356 cm^−1^ are specifically attributed to the A_1_(TO) mode. Also, the results of Ag doping in ZnO coincide with XRD results of secondary phase formations [22–24].

### X-ray photoelectron spectroscopy study of Ag@ZnO nanorods

**Figure 7:**
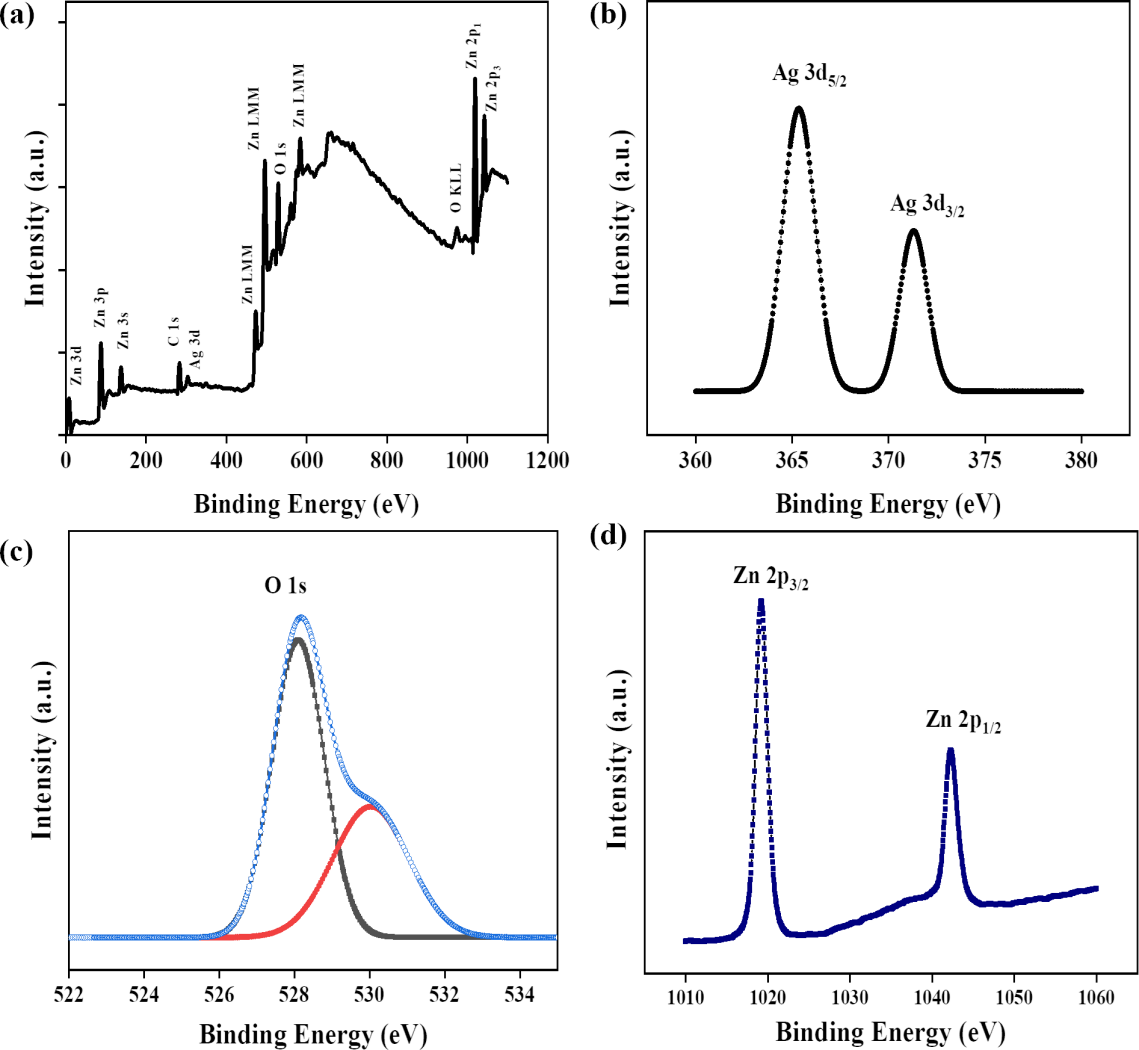
XPS of Ag@ZnO NRs: (a) full scan spectrum, (b) scan of Ag 3d, (c) scan of oxygen 1s, and (d) scan of Zn 2p.

The physical state and chemical compositions of Ag@ZnO NRs were analyzed using X-ray photoelectron spectroscopy (XPS). Figure 7a represents the scan results of the binding energy. The peaks of the curve were attributed to the elements silver, zinc, oxygen, and carbon, whereas no peaks corresponding to other elements were observed. Figure 7b represents the coupled state of the 3d orbital of silver. The peaks at 371.2 and 365.3 eV correspond to Ag 3d_3/2_ and Ag 3d_5/2_, respectively, and the 5.9 eV difference between them indicate that Ag exists in the form of Ag_2_O. This demonstrates the Ag integration into ZnO and the absence of metallic Ag production. The Ag-doped ZnO samples display two distinct peaks at 1042.4 and 1019.2 eV in their Zn 2p spectra (Figure 7d). The peak at 1042.4 eV corresponds to the Zn 2p_1/2_ and the 1019.2 eV peak corresponds to Zn 2p_3/2_. The peak location was marginally changed toward lower binding energy values with the addition of Ag. The peak shift observed was a consequence of the increase in electron density in the host matrix which is caused by the doping of Ag. The 23 eV gap between two binding energies suggests that the Zn element is in a +2 oxidation state. The 1s orbital spectra of oxygen in Ag–ZnO samples are shown in Figure 7c, revealing a peak with a binding energy of 528 eV. The oxygen peak can be resolved into two distinct peaks, which indicates two different types of oxygen species (one at 528 eV due to lattice oxygen of ZnO and another at 530 eV due to presence of surface hydroxy group) [25–26].

### Cyclic voltammetry of modified Ag@ZnO nanorods/gold electrode

**Figure 8:**
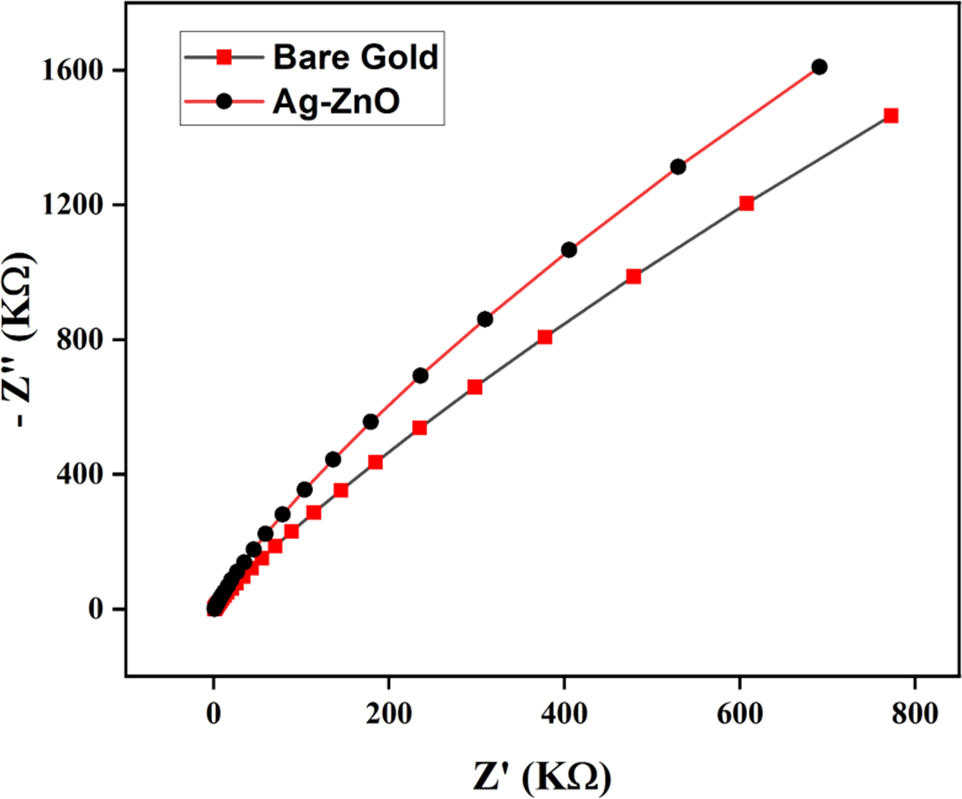
Electrochemical impedance spectroscopy (EIS) Nyquist plots of modified Ag@ZnO NRs/Au and bare Au electrode.

Figure 8 presents the results of an impedance analysis using a frequency-response analyzer (FRA) potentiostat on both fabricated Ag–ZnO/Au electrodes and bare gold electrodes. The modified Ag@ZnO NRs electrode exhibits higher impedance than that of the bare gold electrode. This higher impedance leads to better sensitivity, better electron exchange, and can provide more information about the electrochemical process. The higher impedance of the modified Ag@ZnO NRs electrode improved signal-to-noise ratio, which means that the electrode can better distinguish the electrochemical signal of the analyte from background noise, resulting in more accurate results. Furthermore, a higher impedance of the electrode can provide additional details about the electrochemical process. It can help to understand the kinetics of electron transfer reactions, analyte diffusion, and electrode surface contact mechanisms. A modified Ag@ZnO NRs electrode with greater impedance is more stable and durable. This provides consistent performance throughout time, which is critical for obtaining accurate and repeatable electrochemical results.

**Figure 9:**
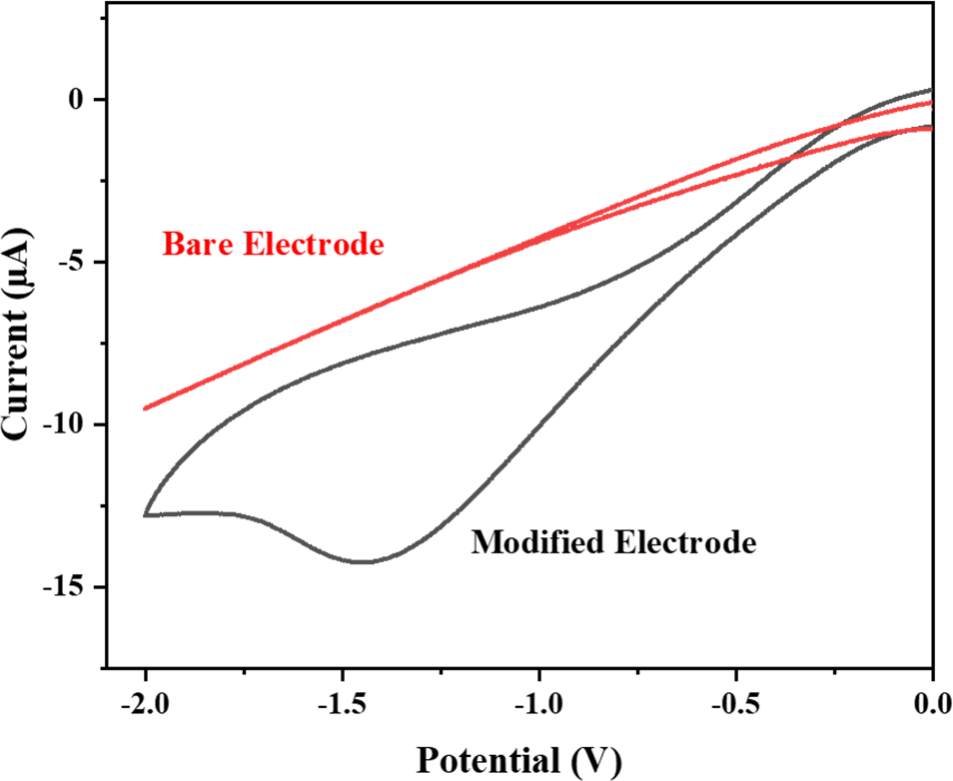
Cyclic voltammetry sweep curves for a modified electrode (black curve) or a bare electrode (red curve) in lead solution.

The lead chemical sensor was fabricated by utilizing the Ag–ZnO nanorods, which functioned as efficient electron mediators. An aqueous solution which includes freshly produced Ag–ZnO nanorods was generated and coated onto a gold electrode. The modified electrode was then dried to form a chemical sensor specifically designed for the detection of lead. The performance of the sensor, including sensitivity, correlation coefficient, and detection limit, was evaluated using cyclic voltammetry. This evaluation involved the usage of a three-electrode system and a gold electrode modified with Ag–ZnO nanorods. The modified electrode was used as a working electrode in the three-electrode system, while the reference electrode was made of Ag/AgCl (sat. KCl), and the Pt electrode was used as a counter electrode [27]. In order to investigate Ag@ZnO NRs response to lead, a standard cyclic voltammetry (CV) experiment was conducted both with and without lead. A typical CV sweep curve for a gold electrode modified with Ag@ZnO NRs in double-distilled water with (black line) and without (red line) lead at a 40 mV/s scan rate is shown in Figure 9.

**Figure 10:**
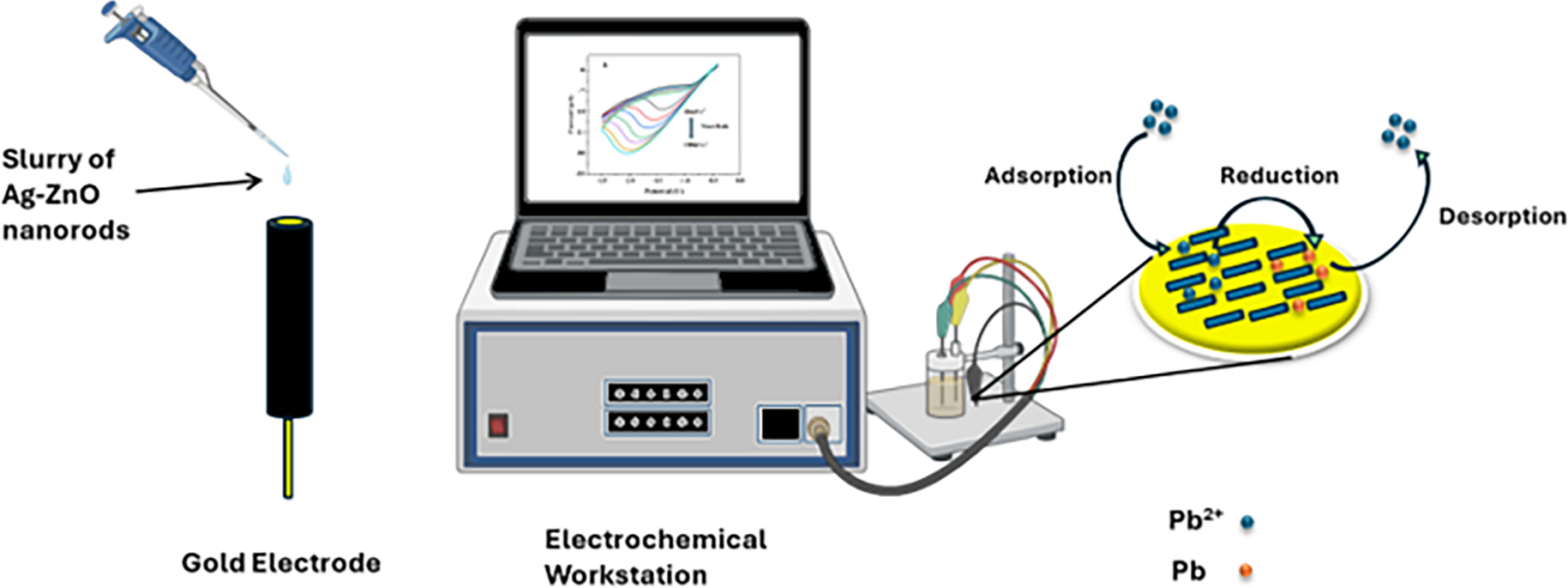
Schematic diagram of the proposed electrochemical detection of lead using a potentiostat and a galvanostat. Figure 10 was partly created in BioRender. Ravinder, R. (2025) https://BioRender.com/g83x775. Afterwards it was adapted with PowerPoint. This content is not subject to CC BY 4.0.

The obtained CV graph demonstrates that the gold electrode modified with Ag@ZnO NRs exhibits no response in the absence of lead (black line). However, when lead is introduced in moderate quantities, a significant reduction peak is distinctly observed in the *I*–*V* graph (red line). A reduction peak appeared approximately at −1.5 V with an *I*_pc_ of −14.5 µA. The obtained *I*–*V* graph clearly shows that the fabricated electrode is highly responsive to lead, which supports the idea that the synthesized Ag@ZnO NRs are useful electron mediators for the creation of lead chemical sensors. The significant rise in peak height is indicative of a faster electron-transfer event because it causes a sharper, more defined peak. Furthermore, the absence of a cathodic current in the reverse cycle indicates the irreversibility of the electrochemical response that was observed [28]. The suggested redox reaction that occurs during the electrochemical sensing experiment is depicted in Figure 10.

### Scan rate study of the modified sensor

**Figure 11:**
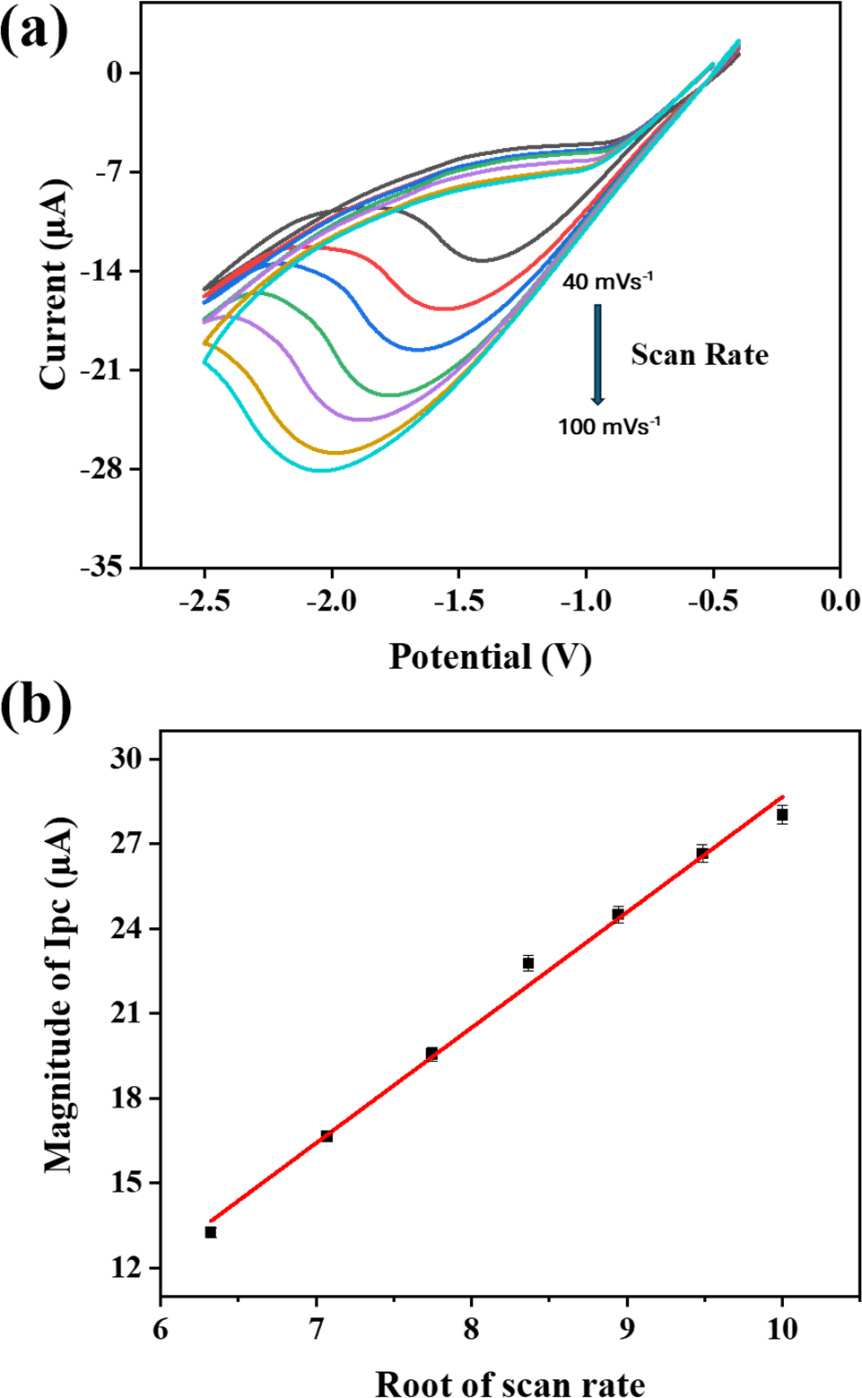
(a) The *I*–*V* response of the modified electrode at varying scan rates and (b) peak current magnitude as a function of the square root of the scan rate of the modified electrode.

Furthermore, by carrying out several scan-rate dependent studies, the electrochemical response of the modified electrode was thoroughly investigated. The studies on scan-rate dependence were conducted at different scan rates between 40–100 mV/s in a solution containing 20 ppm of lead. Figure 11a displays the CV results of the Ag@ZnO NRs/Nafion/gold electrode, using scan rates varying from 40 to 100 mV/s. The observed CV response, which varies with the scan rate, indicates that the current values proportionally increase with higher scan rates, indicating that the reduction process is controlled by diffusion. Figure 11b displays a graph illustrating the relationship between *I*_pc_ (cathodic current) and *v*^1/2^ (square root of the scan rate). Clearly, the cathodic peak current demonstrates a linear connection with *v*^1/2^, demonstrating diffusion-controlled kinetics [29].

### Concentration effect on the peak current or calibration curve

**Figure 12:**
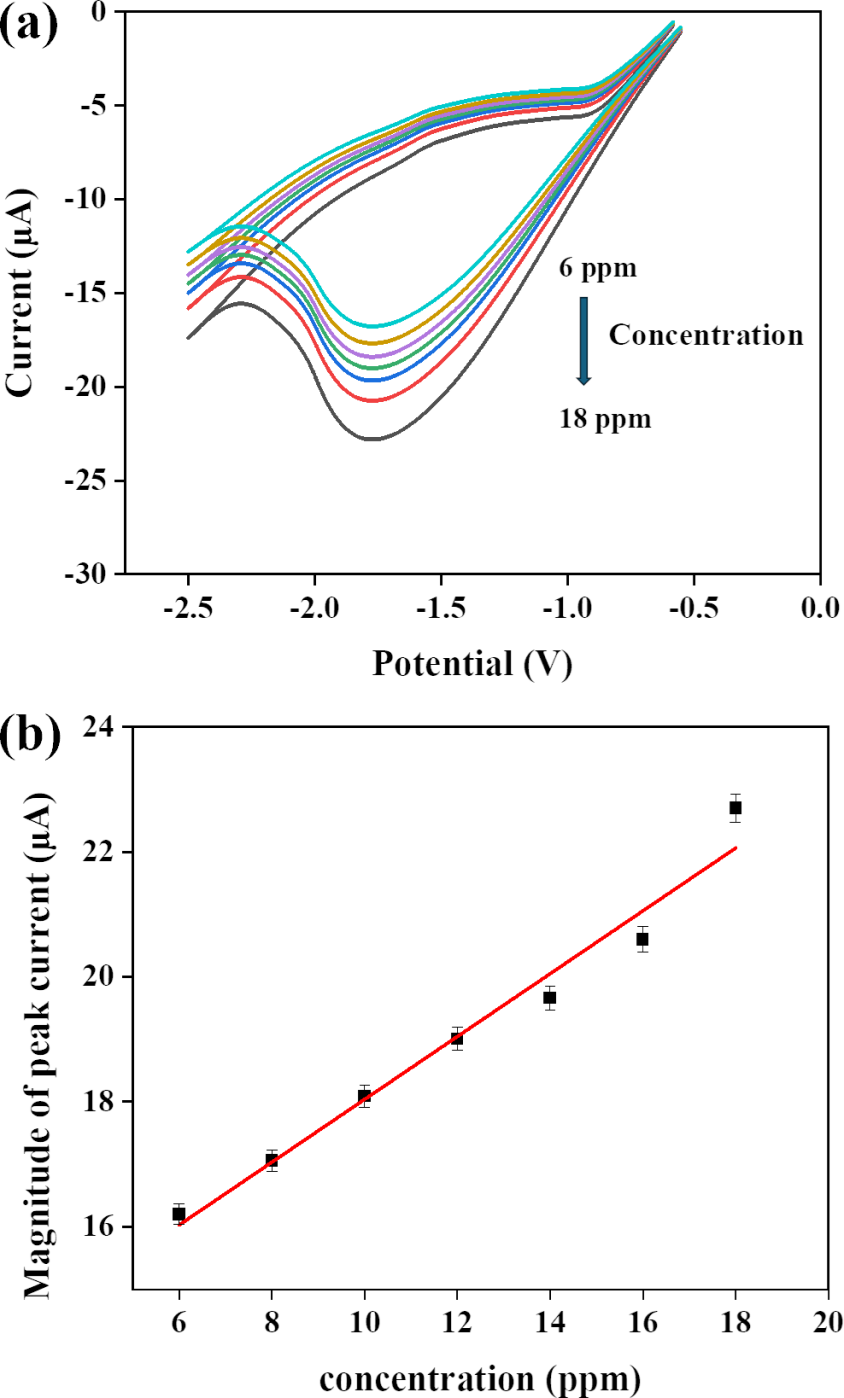
(a) *I*–*V* response of the modified electrode at varying concentrations of lead; and (b) peak current magnitude as a function of lead concentration.

Figure 12a illustrates a steady increase in peak current as lead concentration increases. A standard calibration curve was obtained by graphing the magnitude of peak current data as a function of concentration (Figure 12b). The prepared lead sensor demonstrates a high sensitivity of 16 µA·ppm^−1^·cm^−2^, as determined by dividing the slope of the standard curve with the surface area of the modified electrode. The limit of detection was found to be 3 ppm by estimating it using the standard curve (current as a function of the concentration) illustrated in Figure 12b. The calculation was carried out by dividing the standard deviation by the slope of the standard curve and multiplying by three [30].

### Analytical application of the proposed sensor

**Figure 13:**
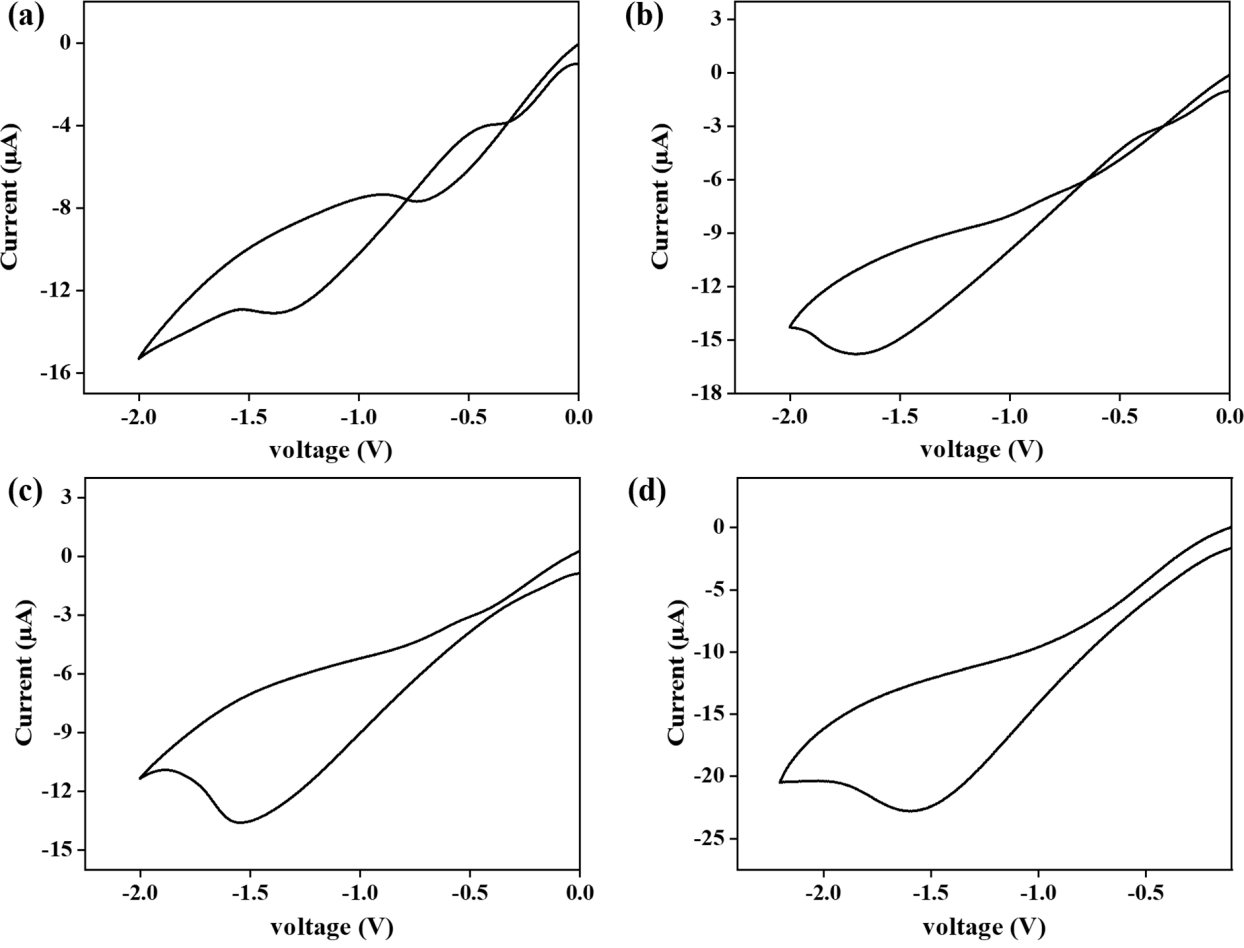
*I*–*V* response of the modified electrode at a varying concentration of lead in (a) canal water, (b) groundwater, (c) water obtained after reverse osmosis (RO) and (d), supply drinking water.

To check the suitability of the proposed lead sensor, different real samples were tested. Real samples were collected from various sources (e.g., tap water, groundwater, canal water, and water contaminated with a known amount of lead). In order to assess the presence of lead, real samples were analyzed by measuring the cyclic voltammetry response of the sensor. As shown in Figure 13, clearly visible signals have been obtained for artificially contaminated samples using the built electrode and the standard approach.

Table 1 presents the samples, the lead spiking amount, and the calculated amount of lead using the aforementioned technique. As the detected amount of lead in real samples are slightly higher than the spiking amount of lead, this is probable due to lead toxicity of real samples or greater conductivity of real samples due to the presence of more ions, which can influence the calculation of lead concentration.

**Table 1:** Quantitative analysis of lead concentrations in real samples artificially contaminated with lead.

Sr. No.	Sample	Amount of lead measured by the modified Ag–ZnO electrode (ppm)	Spiking amount of lead (ppm)

1	spiked canal water	3.0398	3
2	spiked ground water	8.3784	8
3	spiked RO water	4.1752	4
4	spiked supply drinking water	19.5219	19

Table 1 illustrates that the designed sensor and the proposed technique yielded reasonable outcomes. The obtained results are compatible with theoretical calculations, demonstrating the feasibility of the proposed standardized technique. Table 2 presents the comparison of this work with other similar works. The fabricated sensor showed a slightly higher limit of detection (LOD) value, which is due to the use of PBS and the higher impedance of the electrode. An acidic pH provides better sensitivity, but it also brings health and safety issues, environmental impact, can cause electrode corrosion, enhances interference, and is incompatible with real-life or biological samples. Therefore, PBS with pH 7 has been used for electrochemical studies since it provides a stable pH, which is crucial for maintaining consistent electrochemical conditions during experiments.

**Table 2:** Comparison between the fabricated sensor and similar electrochemical sensors.

S. No.	Electrode	LOD	Linear range	Method of detection	Refs.

1	Ag–ZnO modiﬁed glassy carbon electrode	3.5 nM (in acetate buffer)	50–350 nM	DPASV	[18]
2	Cork-modified carbon paste electrode	0.3 µM (in 0.1 M H_2_SO_4_) 4.8 µM (in 0.1 M PBS)	1–25 µM	SWASV	[31]
3	Calcinated and acidified clay-modified carbon graphite electrode	0.15 μM (in 0.1 M Na_2_SO_4_)	0.24–2.6 µM	SWV	[32]
4	CG electrode	0.4 μM (in 0.5 M NaNO_3_)	5–50 μM	ASV	[33]
5	MWCNT+ B18C6 ion-selective electrode	19.9 μM (NaTFPB as a cation exchanger)	0.1–10 mM	potentiometry	[34]
6	Ag@ZnO NRs modified gold electrode	8 μM (3 ppm) (in 0.1 M PBS)	16–48 μM	CV	present work

## Conclusion

In conclusion, a low-temperature co-precipitation technique was utilized to yield highly crystalline nanorods of ZnO doped with Ag. A variety of techniques were employed to analyze the physical and chemical characteristics of as-synthesized Ag@ZnO NRs. The results demonstrate that the synthesized nanorods possess a crystalline structure, specifically a wurtzite hexagonal phase structure (space group: 186: *P*6_3_*mc*) and exhibit advantageous optical properties. The integration of Ag into ZnO nanostructures enhances their optical characteristics and improves their ability to detect heavy metal ions such as lead. The reason for this is that the addition of silver decreases the holes and electron recombination rate, resulting in an expansion of the surface area. Subsequently, they were effectively employed as an electron mediator in the fabrication of highly sensitive lead sensors. The fact that prolonged exposure to lead ions can result in a range of health problems, it is crucial to identify the presence of lead for the sake of public health and environmental preservation. The lead chemical sensor exhibited exceptional sensitivity and a remarkably low detection limit. The synthesized lead sensor had a high sensitivity of 16 µA·ppm^−1^·cm^−2^, along with a detection limit of 3 ppm. Additionally, it demonstrated a response time of less than 2 s. This sensor is most suitable for applications that necessitate cost-effectiveness, rapid readings, and data collection in the field or at the point of care. To our knowledge, this study presents the initial demonstration of a novel method for fabricating an exceptionally responsive lead chemical sensor utilizing Ag@ZnO NRs.

## Experimental Details

### Synthesis of Ag@ZnO nanorods

For the synthesis of Ag@ZnO NRs, chemicals were purchased from Sigma-Aldrich and used as such without any additional purification steps. Firstly, a 2 mL aqueous solution of silver nitrate (AgNO_3_) (0.1 M) and a 100 mL aqueous solution of zinc nitrate hexahydrate (Zn (NO_3_)_2_·6H_2_O) (0.1 M) were simultaneously prepared. After that, the silver nitrate solution was mixed with the zinc nitrate hexahydrate solution. The resulting solution was stirred for approximately 10 min at room temperature. After the reaction time, a 1 M potassium hydroxide (KOH) solution was gradually added drop by drop. This step adjusted the pH of the mixture to 10. Then the mixture underwent further stirring for 2 h at room temperature. Once the stirring was complete, the deposited precipitate was formed. These precipitates were subjected to centrifugation and the pellet was collected; further washing was done multiple times using distilled water and ethanol. The obtained nanoparticles were oven-dried at 70 °C for 2 h. Finally, the nanoparticles were annealed by subjecting them to a temperature of 450 °C in a muffle furnace for 1 h.

### Evaluation of Ag@ZnO nanorods

The Ag@ZnO NRs obtained above were assessed for their morphological, structural, and optical characteristics. The FE-SEM experiments and examination of morphological characteristics were performed on a 7610F Plus/JEOL. A benchtop Miniflex X-ray diffractometer, (Japan model) manufactured by Rigaku, was utilized to analyze the structural and crystal phases. The study was conducted using a wavelength of 1.54 Å and a 2θ angle ranging from 20° to 90°. The elemental compositions were analyzed using EDS and XPS (Model PHI 5000 Versaprobe III). The chemical states were assessed using FTIR spectroscopy with a Perkin Elmer spectrometer model. The spectrum data was acquired between the frequency range of 450 to 4000 cm^−1^ (resolution of 0.5 cm^−1^). The optical characteristics of the obtained Ag-doped ZnO nanorods were determined using UV–visible spectroscopy (Varian Cary-5000) at room temperature. The measurements were taken in the wavelength range of 200 to 600 nm. Raman spectroscopy (Alpha300/WI Tec) was used to investigate the molecular vibrations, rotational energy, electronic energy levels, and scattering characteristics of Ag–ZnO nanorods. The Malvern Nano-ZS90 was utilized to determine the zeta potential of synthesized nanorods.

### Fabrication of the lead sensor / (Ag@ZnO nanorods/gold electrode)

The obtained Ag@ZnO NRs served as an electron mediator for creating a lead electrochemical sensor with high sensitivity. In order to coat the Ag@ZnO NRs, a mixture of 1 mg of as-produced nanorods was mixed in 10 mL of ethanol and sonicated for 30 min. This 4 µL mixture was then applied to the cylindrical gold electrode of 2 mm diameter and allowed to dry at room temperature. Then, 1 µL Nafion solution was applied resulting in a uniform layer of tightly bound nanorods covering the entire electrode surface. Subsequent electrochemical sensing experiments were conducted at room temperature using an Autolab electrochemical workstation with a three-electrode configuration. The working electrode consisted of the modified Ag@ZnO NRs/Au electrode, a platinum wire used as the counter electrode, and the reference electrode was an Ag/AgCl electrode. A 0.1 M phosphate buffer solution with a pH of 7.0 was used for all measurements.

## Data Availability

Data generated and analyzed during this study is available from the corresponding author upon reasonable request.
